# Online Sexual Harassment Perpetration Among Peer Adolescents: A Cross-National and Cross-Gender Study

**DOI:** 10.3390/bs15070969

**Published:** 2025-07-17

**Authors:** Estrella Durán-Guerrero, Annalaura Nocentini, Ersilia Menesini, Virginia Sánchez-Jiménez

**Affiliations:** 1 Department of Developmental and Educational Psychology, Universidad de Sevilla, 41013 Sevilla, Spain; eduran1@us.es; 2 Department of Education, Languages, Intercultures, Literatures and Psychology, Università degli Studi di Firenze, 50135 Florence, Italy; annalaura.nocentini@unifi.it (A.N.); ersilia.menesini@unifi.it (E.M.)

**Keywords:** online sexual harassment, perpetration, adolescence, cross-national study, cross-gender study, non-consensual sharing of sexual content

## Abstract

This study aims to validate the Online Sexual Harassment Perpetration among Peers (OSHP-P) instrument for assessing online sexual harassment among adolescents in two different countries, Spain and Italy, considering both new forms of online sexual harassment and gender differences. The instrument was validated by means of a Confirmatory Factor Analysis (CFA) with a sample of 1041 Spanish (*M_age_* = 15.0, *SD* = 0.88) and 1385 Italian (*M_age_* = 14.8, *SD* = 0.87) adolescents, demonstrating factorial invariance across both country and gender. The best-fitting model was two-dimensional, with ambiguous and direct Sexual Cyber Perpetration (SCP) and Non-Consensual Sharing Perpetration (NCSP) factors. Co-involvement (i.e., involvement in both types of aggression) rates were 10.3% in Spain and 7.8% in Italy. No significant gender differences were found for involvement in either the overall scale (46.4% for girls, 44.1% for boys) or the NCSP subscale (3.0% girls vs. 2.2% boys), although significantly higher co-involvement was found among boys (7.7% girls vs. 10.1% boys). This study contributes to the existing body of research on online sexual harassment among peers in adolescence by presenting a new assessment tool that has been shown to be invariant between Spanish and Italian adolescents, as well as between boys and girls.

## 1. Introduction

During adolescence, both boys and girls are faced with the developmental task of building and developing their sexual identity. Through interaction with their peers, adolescents learn about and discover the limits of their behaviours and explore their sexual interests and tastes (Furman & Rose, 2015). Within these dynamics, social networking sites have emerged as significant allies, enhancing communication and flirtation among peers, along with the exchange of sexual content and self-exploration (Mori et al., 2022). The accessibility and perceived anonymity inherent to digital contexts offer adolescents an apparently ‘safe’ environment in which to express and explore their sexuality (Van Ouytsel et al., 2019). However, this anonymity may also be used to perpetrate aggressive behaviour (Marinoni et al., 2024), including sexual behaviours that can lead to online sexual harassment (Madigan et al., 2018).

In the present study, online sexual harassment among adolescents is defined as any sexual attention or behaviour, engaged in using electronic devices, which is non-consensual and unwanted by the victim, thereby causing distress or discomfort (Worsley & Carter, 2021). Such behaviour is a global concern, with prevalence rates of perpetration ranging from 2.7% to 70% (Patel & Roesch, 2022; Penado et al., 2019). This high level of variability in prevalence rates is primarily attributed to the wide range of different ways in which the phenomenon is defined and operationalised (i.e., the dimensions and forms of aggression considered) in scientific studies (Franceschi et al., 2023; Patel & Roesch, 2022). Indeed, although the literature conclusively states that online sexual harassment is a multidimensional phenomenon that encompasses several interrelated forms of aggression, there is no single model that encapsulates all of its specific manifestations (Barak, 2005; Fitzgerald et al., 1995; Henry & Powell, 2018).

### 1.1. The Conceptualisation of Online Sexual Harassment

Initial conceptualisations of online sexual harassment were models derived from those that had been developed for face-to-face sexual violence, such as the one proposed by Fitzgerald et al. (1995), who differentiated between the following three main forms: gender-based harassment, sexual coercion, and unwanted sexual requests. Drawing on this model, Barak (2005), for instance, emphasised the influence of the online context on online sexual assaults, differentiating between direct and passive forms. Whereas direct assaults target a specific victim, the synchronicity and detachment of online environments also encourage assaults that lack any immediate or explicit target, inflicting harm in a more indirect or ambiguous (passive) fashion. Conversely, McGlynn and Rackley (2017) argued for the importance of exploring new forms of sexual violence that are specific to the online context under the label image-based sexual abuse (IBSA). In this case, the authors sought to focus attention on the aggressive nature of such conduct, advocating for the label as a replacement for revenge porn. The term encompasses the non-consensual distribution and creation of private sexual images, sextortion, online voyeurism, and ‘non-consensually created sexual images involving the recording of rapes or other forms of sexual assault’ (McGlynn & Rackley, 2017, p. 439). More recently, Henry and Powell (2018) introduced the term ‘technology-facilitated sexual violence’ (TFSV) to emphasise the different behaviours that could be carried out thanks to the use of technology. The authors differentiate between ‘online/digital sexual harassment, gender- and sexuality-based harassment, cyberstalking, image-based sexual exploitation, and the use of a carriage service to perpetrate a sexual assault or coerce an unwanted sexual experience’ (Henry & Powell, 2018, p. 196).

### 1.2. Gaps in the Model

The aforementioned conceptualisations of sexual harassment are still emerging and have some limitations that are worth mentioning. First, some models describe types of sexual violence that have been directly transposed from the face-to-face context to the online one (Walters & Espelage, 2020), without considering the specific ways in which the online context fosters the development of new forms of harassment (Barak, 2005). Second, even though some current models do indeed consider these new forms, studies tend to focus solely on a few manifestations (McGlynn & Rackley, 2017), overlooking others that are highly relevant during adolescence such as those associated with gender or sexual diversity (Espelage et al., 2022). Moreover, most studies focus on adult populations (Henry & Powell, 2018), describing dimensions (e.g., ‘the use of a carriage service to perpetrate a sexual assault or coerce an unwanted sexual experience’) that are not particularly representative of the adolescent population.

This variability in the conceptualisation of different types of online sexual harassment is further compounded by the scarcity of validated instruments. Existing measures focus primarily on online sexual victimisation, with few instruments to date analysing perpetration among adolescents (Franceschi et al., 2023). Following Barak’s (2005) model, Sánchez-Jiménez et al. (2017) adapted the measure developed by the American Association of University Women (2001), validating the instrument with a sample of Spanish adolescents using both exploratory factor analysis (EFA) and confirmatory factor analysis (CFA) and demonstrating factorial invariance across gender. Two dimensions were identified, direct and passive aggression, although due to the high level of correlation between them, the authors recommended they be amalgamated into a single dimension scale. Gámez-Guadix and Incera (2021) validated some of the dimensions proposed in the model developed by Henry and Powell (2018) for online sexual harassment victimisation among adolescents using an EFA. However, in this case these dimensions were not confirmed by a CFA, and the correlations between the latent factors were not indicated (Franceschi et al., 2023). In a subsequent study using network analysis (Gámez-Guadix et al., 2022b), the authors confirmed an overlap between different forms, with gender- and sexuality-based victimisation forming a cluster with digital sexual harassment victimisation, and sextortion and non-consensual pornography forming a second cluster. These studies highlight two important ideas. First, they indicate that during adolescence different forms of sexual harassment correlate strongly with each other. And second, they suggest that gender- and sexuality-based sexual harassment and other direct verbal forms differ from more severe forms, such as sexual threats and the non-consensual distribution of sexual content, at least in terms of victimisation.

In this regard, a meta-analysis by Patel and Roesch (2022) found that the distribution or forwarding of unwanted and non-consensual sexual content was one of the most prevalent new forms of adolescent online sexual harassment. Non-consensual sharing of sexual content (NCS; Walker et al., 2021) refers to all messages, images, and videos of a sexual nature that are sent to another person without the victim’s consent (Walker & Sleath, 2017), with recent meta-analyses indicating a prevalence of 14.5% (Mori et al., 2022). The literature on the phenomenon has gradually increased over recent years, with studies using different labels such as non-consensual sexting/pornography, revenge porn, or non-consensual forwarding sexts (Barroso et al., 2023; Gámez-Guadix et al., 2022b; Madigan et al., 2018; Maes et al., 2023). Measurement of this phenomenon is also controversial, and, to date, few studies have analysed its relationship with other forms of online sexual harassment during adolescence (Gámez-Guadix et al., 2022a).

In light of the above, it is important to analyse NCS within the construct of online sexual harassment. This would enable NCS (as a specific form of online sexual harassment) to be compared with traditionally studied forms and would promote the analysis of its prevalence among adolescents at an international level.

### 1.3. Cross-National Considerations

Online sexual harassment has attracted increasing attention in Europe over recent years, as evidenced by a recent systematic review of 32 European studies on the phenomenon (Franceschi et al., 2023). However, the majority of these studies focus on victimisation, with only five studies in Europe (Spain, Italy, and Belgium) specifically focusing on the perpetration of online sexual harassment during adolescence.

Despite growing interest, however, there are still few instruments that have been validated across different countries with the aim of enabling reliable prevalence comparisons. Studies that enable the cross-national validation of measures are vital to advancing our understanding of the phenomena, as they promote the development of equivalent measures sensitive to the differences that exist between countries.

Spain and Italy are Mediterranean countries in Western Europe that are fairly similar in terms of language and cultural–social norms. Both countries also have a long tradition of research into interpersonal violence during childhood and adolescence (Taiti et al., 2024; Cabrera-Vázquez et al., 2025), and studies on online sexual harassment have multiplied recently (e.g., Crapolicchio et al., 2022; Cricenti et al., 2022; Díaz-Aguado & Martínez-Arias, 2022; Durán-Guerrero & Sánchez-Jiménez, 2025; Franceschi et al., 2024; Martínez-Bacaicoa et al., 2024; Miguel-Álvaro et al., 2024; Morelli et al., 2020). An initial comparative study between Spain and Italy on face-to-face sexual victimisation in girls, conducted in 2010, found that, in Spain, the prevalence of visual/verbal forms was twice as high as in Italy, whereas Italian girls were more involved in visual/verbal and physical forms than their Spanish counterparts (Ortega et al., 2010). In terms of online sexual harassment in Spain, Sánchez-Jiménez et al. (2017) found rates of around 25% for online sexual victimisation, whereas in Italy, Franceschi et al. (2024) found rates of over 40%. For NCS in Spain, the perpetration rate ranges between 4.9 and 14.3% (Durán-Guerrero & Sánchez-Jiménez, 2025; Gámez-Guadix et al., 2022a); whereas in Italy, the figure stands slightly higher at 18.3% (Morelli et al., 2020). To the best of our knowledge, no studies to date have directly compared the two countries in terms of online sexual harassment or, more specifically, the perpetration of online sexual harassment among peers during adolescence.

### 1.4. Online Sexual Harassment and Gender

Gender constitutes a pivotal variable in the study of interpersonal violence, particularly in relation to sexual harassment. Studies suggest that boys and girls perceive and experience this type of violence differently, with a more pronounced emotional impact being observed in girls than in boys (L. Reed et al., 2017). However, studies on gender differences in sexual aggression have so far yielded inconclusive results. Mori et al. (2022), for instance, found no gender differences in the perpetration of different forms of online sexual harassment among adolescents. Conversely, a meta-analysis conducted by Patel and Roesch (2022) found a notably higher prevalence of specific forms of online sexual harassment perpetration among boys (4.7% vs. 3.6%). Some authors suggest that this lack of consensus may stem from a paucity of studies specifically concentrating on perpetration (Buchanan & Mahoney, 2022; Franceschi et al., 2023), and advocate for more studies that advance this avenue of research by developing measures capable of capturing gender differences in this field.

### 1.5. The Present Study

The analysis of online sexual harassment during adolescence is still an emerging field of study that requires validated instruments in order to make headway in the following four different areas: Firstly, in the determination of its specific forms or typologies. Previous theoretical models suggest that traditional forms of harassment (transposed directly from the offline to the online environment) are interrelated in the online context (Barak, 2005), at least in terms of victimisation (Sánchez-Jiménez et al., 2017). Nevertheless, few studies to date have included new, and more serious, online-specific forms of sexual harassment (Martínez-Bacaicoa et al., 2024) such as NCS. Secondly, validated instruments are needed to examine the perpetration of online sexual harassment among adolescents in greater depth. To date, most studies have focused on victimisation, with few focusing on perpetration or aggression. Thirdly, validated instruments are needed to enable results to be compared across countries. The scarcity of studies and measures makes it difficult to do this at present, preventing any adjusted estimation of these phenomena in the adolescent population. Finally, in light of the above, further studies are required that take gender differences in perpetration into account, identifying both similarities and differences between girls and boys in relation to different forms of online sexual aggression.

The present study aims to further this avenue of research by adapting and validating an instrument designed to assess the perpetration of online sexual harassment among adolescents in two different countries, Spain and Italy, taking new forms of online sexual harassment into account and considering gender differences. The starting point for the adaptation was the Peer Sexual Cyber-Victimisation Scale (SCV; Sánchez-Jiménez et al., 2017), a Spanish adaptation of the instrument developed by the American Association of University Women (2001) that has also been validated for measuring sexual cyber victimisation in Italy (Franceschi et al., 2024). Since the SCV has been found to have good psychometric properties when used in adolescent populations in both countries, it was deemed suitable for adaptation to perpetration. However, the present study also aims to incorporate new specific forms of online sexual harassment that are relevant during adolescence, such as NCS, in the belief that this will provide a fuller picture of the diversity and complexity of behaviours presently manifesting in digital environments and enable a more comprehensive empirical assessment of both boys and girls.

Therefore, the aims of the present study are as follows: (1) to confirm the factor structure of the Peer Sexual Cyber-Victimisation Scale instrument adapted to measure perpetration in the adolescent population and encompassing a new NCS dimension, which we will call the Online Sexual Harassment Perpetration among Peers scale; (2) to analyse the factorial invariance of the Online Sexual Harassment Perpetration among Peers scale across Italy and Spain; (3) to examine the factorial invariance of the scale in terms of gender.

## 2. Materials and Methods

### 2.1. Participants

The sample was made up of 2426 secondary school students from Andalucía (southern Spain) and Toscana (central Italy). The Spanish sample (*n* = 1041) was recruited from public secondary schools in Seville and Huelva (47.4% defined themselves as girls, 50.9% as boys, 0.9% both, and 0.9% neither). The age range was 12–18 years (*M_AGE_* = 15.0, *SD* = 0.88). The Italian sample (*n* = 1385) was recruited from public and private secondary schools in Florence, Lucca, Massa Carrara, Pistoia, Pisa, Livorno, Arezzo, and Prato (50.9% defined themselves as girls, 45.7% as boys, 0.3% as other, and 3.0% preferred not to answer). The age range was 12–18 years (*M_AGE_* = 14.8, *SD* = 0.87).

### 2.2. Procedure

The Spanish sample formed part of the first wave of two Spanish state-wide projects. The education authorities provided a random list of schools, and six public secondary schools agreed to participate in the study. These schools were contacted and informed of the objectives and conditions for participating in the project. The same information was also sent to families, along with an informed consent document for their children to participate in the study. Authorised students completed the questionnaire in the classroom during school hours in 2022. The students were informed of the study aims, the voluntary nature of their participation, and the confidentiality of the data collected. The study was approved by the Ethics Committee of the University of Seville (codes: 0606-N-22 and 2024-2709).

The Italian sample included adolescents coming from two projects. The first one is a longitudinal project called ‘Prejudicial bullying involving ethnic groups: Understanding mechanisms and translating knowledge into effective interventions’. The second one is the control group of an antibullying intervention programme called NoTrap!, which is implemented every year in Tuscany. Both projects are science-based Italian initiatives focused on preventing and reducing (cyber)bullying among children and adolescents. They seek to understand the causes of bullying and develop evidence-based interventions. A total of 60 secondary schools were contacted by email and asked to collaborate in the projects, and 21 expressed an interest in participating, with at least four classes in each school. Student consent was requested for participants aged 14 years and over, and parental consent was requested for those under 14 years of age. Data were collected during school hours in 2022, and students were reassured of the confidential nature of the information they provided and the voluntary nature of their participation. This study was approved by the Catholic University Committee (protocol n. 37-20 and 87-21) and by the University of Florence Ethics Committees for Research (code: 0173384).

Spanish participants completed the questionnaires in pencil and paper format during school hours. Qualified researchers were in the classrooms to assist participants when required. Italian participants completed the questionnaires online, also during school hours, with qualified researchers connected remotely. Previous studies highlight the no differences between the use of paper–pencil or online formats (e.g., Dodou & de Winter, 2014; Gwaltney et al., 2008). The Italian questionnaires were translated from the Spanish and original version through a highly fluent researcher. A coordination meeting was also organised to resolve any discrepancies and ensure semantic equivalence. Subsequently, both the Spanish and Italian questionnaires were translated into English to ensure accuracy.

### 2.3. Instruments

Sociodemographic information: Gender and age were measured. Gender was measured as boy, girl, binary, non-binary, other, and ‘*I’d rather not answer*’.

Online Sexual Harassment Perpetration among Peers (OSHP-P): The instrument comprised two scales which were adapted to present a homogeneous linguistic style.

(1) The Peer Sexual Cyber-Victimisation Scale (SCV; Sánchez-Jiménez et al., 2017) was modified and adapted to measure perpetration. The scale comprises the following two subscales: 4 items for Ambiguous Sexual Cyber Perpetration (ASCP) and 5 items for Direct Sexual Cyber Perpetration (DSCP). The ASCP included items referring to ambiguous sexual exchanges such as ‘Making sexual comments, jokes, or gestures on social networking profiles or via WhatsApp’, and the DSCP included items associated with explicit attacks or requests directed at a specific person, such as ‘Making jokes or spreading false rumours about someone’s sexual behaviour on their social media profile or via WhatsApp’. Students were asked to indicate the frequency with which they had engaged in these behaviours over the last two months when the other person did not want to, using a 5-point frequency scale (1 = never, 2 = once or twice, 3 = more than twice per month, 4 = every week, 5 = every day).
(2) The Non-Consensual Sharing Perpetration (NCSP) scale is an adapted and reduced version of the Sex and Tech questionnaire used by Walker et al. (2021). It comprises 3 items rated on a 5-point frequency scale (1 = never, 2 = once or twice, 3 = more than twice per month, 4 = every week, 5 = every day) that assess how often the respondent has forwarded messages, images, and videos of another person without their consent. Participants were asked to state how often they had engaged in a variety of sexual harassment behaviours during the last 2 months. An example of an item would be ‘Forwarding intimate messages from other people without their consent’.

### 2.4. Analysis Plan

Frequency, skewness, and kurtosis were assessed. Due to the strongly asymmetric distribution of the items, they were dichotomised into 1 = no and 2 = yes. The prevalence and distribution of items in both countries are displayed in Table 1.

To address the first aim, a CFA was performed to examine the instrument’s factor structure. Two models were tested. The first was a three-factor first-order model following the instrument’s original scales (ASCP + DSCP + NCSP). The second consisted of two first-order factors, combining the ambiguous and direct perpetration subscales into a single factor (ASCP + DSCP = SCP) with NCSP as the second factor (SCP + NCSP). After selecting the model with the best fit indices, the internal consistency of the factors was analysed. The selected model was tested in each country. The weighted least square mean and variance adjusted estimator and theta parameterisation (WLSMV; Brown, 2006; Muthén & Muthén, 2012) were used due to the categorical nature of the data. The root mean square error of approximation (RMSEA) and comparative fit index (CFI) were used to assess goodness of fit. The cut-off points were <0.06 for RMSEA and >0.90 for CFI (Kenny & McCoach, 2003).

To respond to the study’s second aim, invariance analyses by country were performed in two steps, starting with a less restrictive model before moving on to a more restrictive one. In the first step, configural invariance was tested, with free factor loadings and item thresholds, item residuals set to 1, factor means set to 1, and variance set to 0 in each group. In the second step, strict invariance was tested, with factor loadings and item thresholds set to be the same in both groups, residual variances set to 1 in the first group (free in the second group), factor means set to 0 in the first group (free in the second group), and variance set to 1 for identification in both groups. The WLSMV estimator and theta parameterisation were also used. Chi-square test values were reported; however, they were not used in the model comparison due to their sensitivity to large sample sizes (Kline, 2015). Instead, the variation in two alternative fit indices to χ^2^ in the invariance analysis was analysed (Rutkowski & Svetina, 2014). The selected fit indices were again RMSEA and CFI, with only one difference: <0.015 for ∆RMSEA and <0.010 for ∆CFI (Chen, 2007).

To achieve the third aim and test gender invariance, the decision was made to remove the non-binary, other, and neither groups from the analysis due to their low prevalence (*n* = 66, 2.7%). Consequently, invariance between girls and boys was tested following the previously reported steps. Analyses were performed with Mplus 8.3 and SPSS 29.

## 3. Results

**Figure 1:**
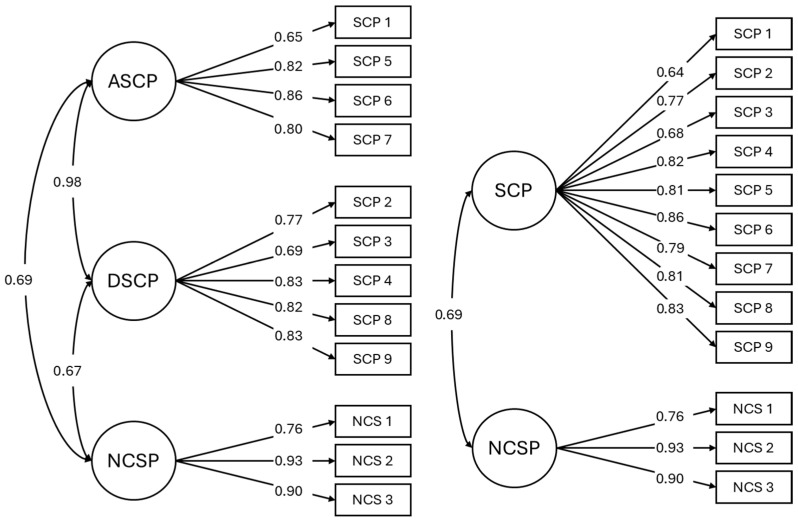
Confirmatory factor analysis models: structure and standardised factor loadings.

To respond to the study’s first aim, a CFA was performed to test the factor structure of the two models in the total sample. Model 1 comprised three factors; whereas Model 2 comprised two factors (see Table 2). Factor loadings and structure of both models were included in Figure 1. Both models were found to have adequate fit indices. However, very high standardised correlations were observed between the ASCP and DSCP factors of Model 1 (*r* = 0.98), whereas Model 2 returned adequate correlations between its two factors, *r* = 0.69. Consequently, Model 2 was deemed to fit the data better, with an average variance extracted (AVE) of 0.61 for SCP and of 0.75 for NCS, and a composite reliability (CR) value of 0.93 for SCP and of 0.90 for NCS. Reliability analyses of the resulting factors were performed in each country, with optimal results being obtained for both Spain (SCP, Ω = 0.86; NCSP, Ω = 0.75) and Italy (SCP, Ω = 0.80; NCSP, Ω = 0.80).

To address the second aim, CFAs were conducted in both Spain and Italy with the two-dimensional model as a preliminary step prior to the invariance analyses (see Table 2), with excellent fit indices being found in both countries. In the cross-national invariance analyses (Table 3), the configural step returned excellent fit indices. Standardised results were also excellent in both countries, with all factor loadings (estimates exceeded 0.50), intercepts, and thresholds being significant. The strict step also returned excellent results. The analysis of differences between the fit indices of the configural and strict steps did not return poorer results, thereby indicating strong factorial invariance across countries. Achieving strong factorial invariance made it possible to compare prevalence rates between countries.

Finally, cross-national invariance enabled us to explore cross-gender invariance, thereby responding to the study’s third aim. The CFA results in boys and girls (included in Table 2) revealed adequate fit indices. Invariance analyses also returned optimal results at baseline (see Table 3). The configural step indicated excellent fit indices. Standardised results were also excellent for both genders, with all factor loadings (estimates exceeded 0.50), intercepts, and thresholds being significant. Next, the strict step was performed, with excellent fit indices being obtained once again. When the two steps were compared, the restriction of the fit indices was not observed to result in poorer values, thereby indicating strong factorial invariance between boys and girls. Achieving strong factorial invariance enabled the comparison of prevalence rates between boys and girls.

Table 4 presents the prevalence results by country and gender. Significant differences were observed between countries [χ^2^(3)= 11.92, *p* < 0.01], although with a small effect size (Cramer’s *V* = 0.07). Spain had significantly higher prevalence rates in the overall scale (OSHP-P: 48.3% vs. 42.9%, *p* < 0.01), as well as for involvement in NCSP only (3.5% vs. 2.0%, *p* < 0.05) and co-involvement in both forms of online sexual harassment (10.3% vs. 7.8%, *p* < 0.01). Both countries had higher prevalence rates for involvement in SCP only (Spain: 34.5% vs. Italy: 33.1%), with no significant differences between them.

The gender analyses revealed significant differences between boys and girls [χ^2^(3) = 8.25, *p* < 0.05], albeit with a small effect size (Cramer’s *V* = 0.05). Girls reported significantly higher prevalence rates for involvement in SCP only (girls: 35.6% vs. boys: 31.8%, *p* < 0.01), whereas boys scored significantly higher for co-involvement in both forms of online sexual harassment (girls: 7.7% vs. boys: 10.1%, *p* < 0.01). No gender differences were found in involvement rates in either the overall scale (OSHP-P: girls: 46.4% vs. boys: 44.1%) or the NCSP subscale (girls: 3.0% vs. boys: 2.2%).

## 4. Discussion

The present study aimed to validate the Online Sexual Harassment Perpetration among Peers (OSHP-P) instrument by adapting the SCV scale (Sánchez-Jiménez et al., 2017) to perpetrators (SCP) among Spanish and Italian adolescents and adding NCS perpetration as a new dimension. The results indicated that the best-fitting model was two-dimensional, with combined ambiguous and direct sexual harassment perpetration in one factor (SCP) and NCS perpetration in another (NCSP). This model was validated among Spanish and Italian adolescents, indicating that the OSHP-P is a valid international measure for evaluating online sexual harassment perpetration among peers in adolescence. These results enable the achievement of the study’s first objective. Similarly to that found in the study by Sánchez-Jiménez et al. (2017) on online sexual victimisation in adolescence, the direct and ambiguous dimensions identified by Barak (2005) seem to overlap in the online context also. In other words, insulting someone because of their sexual orientation on social media (direct aggression) does not seem to differ too much from posting sexual comments on networking sites (passive aggression). Similar results were found also by Gámez-Guadix and Incera (2021) in their study on online victimisation. The close correlation between the two forms may be explained by adolescents’ developmental stage, in which the exploration and construction of their sexual identity is a key developmental task. Their urge to accomplish this task may increase their interest in exploring sexuality, which in turn may prompt them to engage in behaviours in which the boundaries between consented and bothersome conduct become blurred, leading to harassment. Combined with the disinhibition effect of the online environment (Suler, 2004) and the lack of awareness of the consequences of their actions many adolescents demonstrate in online acts of aggression (Barrense-Dias et al., 2020; Cohen-Almagor, 2018), this may explain the high prevalence rates found in this dimension, with one in two adolescents in both countries claiming to have engaged in such actions over the last two months. The validation of a two-dimensional factor structure confirmed that forwarding sexual content without consent is indeed a relevant dimension of online sexual harassment among adolescents. In this case, the strength of the correlation between the two factors suggests that both forms, while related, have a separate identity, with NCS emerging as a specific form of online sexual harassment recognised by adolescents (Franceschi et al., 2024; Patel & Roesch, 2022).

The invariance analysis confirmed the validity of the structure across countries and genders, meaning that the scale can be used in both countries and to reliably compare prevalence rates between boys and girls, enabling the achievement of the study’s second and third objectives. These results make an important contribution to the field of study. Firstly, the between-country invariance found confirms that Spanish and Italian adolescents have the same understanding of online sexual harassment, both in the traditional face-to-face verbal/visual forms studied (including gender-based harassment, homophobic online harassment, and unwanted sexual attention) and in the specific forms. We can therefore conclude that the differences observed between countries are due to variations in prevalence rates, not discrepancies in participants’ interpretation of the instrument (Putnick & Bornstein, 2016; Vandenberg & Lance, 2000). These similarities are partly attributed to social and cultural factors that are common to both these Mediterranean countries, which have traditional gender patterns that support and legitimise sexist or aggressive behaviour among peers (Jewell et al., 2015; Leaper & Brown, 2018). Furthermore, the Spanish and Italian education systems have similar institutional structures and dynamics and face similar significant challenges in terms of regulating the online environment and preventing sexual harassment among peers (Rodríguez-deArriba et al., 2025; Rossini & Peiró-i-Gregòri, 2015). Previous cross-national Italy–Spain studies have already confirmed the validity of the factor structure in other scales measuring, for example, adolescent dating violence (Menesini et al., 2011) and sexual victimisation among girls (Ortega et al., 2010). The present study confirms that young people’s perceptions of online sexual harassment are similar also in the two countries.

In relation to gender, the study results indicate that the scale is invariant for boys and girls. Gender invariance is crucial in psychometric and social research, especially when developing measures that assess constructs so directly related to gender, such as intimate partner violence and adolescent sexual harassment (Gámez-Guadix et al., 2022b; E. Reed et al., 2020; Powell & Henry, 2019; Walker et al., 2021). If a measurement instrument is not gender-invariant, the differences found could reflect measurement bias effects rather than real differences between groups (Kline, 2015). For sexual harassment, not all studies have achieved gender invariance. Shorey et al. (2019) attempted to achieve metric invariance between boys and girls in the perpetration of teen dating violence, but were unsuccessful, with the results indicating low internal consistency in the sexual abuse subscale. In contrast, Vega-Gea et al. (2016) did manage to achieve it for face-to-face peer sexual harassment perpetration. The present study contributes to this avenue of research by providing a cross-gender invariant instrument for measuring online sexual harassment perpetration in two countries.

Achieving factorial invariance enables the comparison of online sexual harassment perpetration rates across countries and genders. In general, adolescents reported greater involvement in direct and ambiguous forms than in NCS in both Spain and Italy, with co-involvement in both forms being 10.3% in Spain and 7.8% in Italy. These data suggest that, in both countries, forwarding sexual content featuring someone without their consent is a form of harassment that, when it occurs, is often associated with other more frequent and normalised forms of online sexual aggression (Clancy et al., 2019; Gámez-Guadix et al., 2022b; Ybarra & Thompson, 2018), such as the direct and ambiguous forms measured in this study. Moreover, these data may also indicate that NCS behaviours are perceived by adolescents as more serious than other more frequent behaviours (e.g., talking about sex when the other person does not want to), as they violate the victim’s privacy and damage their reputation (L. Reed et al., 2020; Lucero et al., 2014; Sánchez-Jiménez et al., 2022) and tend to appear only once other forms of harassment have already been established. NCS data are worth noting, with prevalence being approximately 14% and 10% in Spain and Italy, respectively. These figures are consistent with those reported in other international studies (Mori et al., 2022), although they are slightly lower than those found by other studies conducted in Italy (Morelli et al., 2020).

The between-country prevalence analysis revealed higher levels of perpetration among Spanish adolescents than among their Italian counterparts, with significant differences (although a small effect size) in relation to involvement in NCS alone and co-involvement in both forms analysed. Although no previous studies have sought to compare Spain and Italy in terms of online sexual harassment, the data point to a higher overall level of engagement among Spanish adolescents than among Italian ones, a finding that is consistent with those reported by previous studies on face-to-face sexual harassment (Ortega et al., 2010). However, studies focusing on online sexual victimisation have observed opposite trends, with Italy reporting higher prevalence rates (Franceschi et al., 2024) than Spain (Sánchez-Jiménez et al., 2017). The time gap between these studies may have influenced the results, given the changing nature of adolescent social media use. Additionally, it should be noted that the samples used in both studies are not fully representative of national data, which may further limit the comparability of the findings. The limited effect size found in this study prevents us from drawing any firm conclusions about the differences in prevalence rates between countries, and further studies are required to confirm or refute these results.

In the gender prevalence analysis, girls reported slightly higher engagement rates in both forms of online sexual harassment, but not in co-involvement, in which the prevalence rate among boys was higher. Even though these results must be taken with caution due to the very small effect size, they nevertheless indicate that it is more likely for boys to engage in both forms of online sexual harassment simultaneously, whereas girls are more likely to engage in a single form. Several previous studies have pointed out that these results may be explained by gender differences in sexual experimentation (Vandenbosch, 2020), with boys feeling freer than girls to develop and explore their sexuality (Cava et al., 2020), prompting them to engage in more online sexual harassment (Gámez-Guadix et al., 2022b). However, other authors argue that this differential prevalence may indicate different motivations for harassment among boys and girls (Barrense-Dias et al., 2020; L. Reed et al., 2020). Again, further studies are required to clarify this issue.

### Limitations and Future Research

This study significantly advances our knowledge in the field of online sexual harassment among peers in adolescence, presenting a new measure of the phenomenon that is invariant across both country and gender. However, the study also has certain limitations, the first being the composition of the ambiguous and direct sexual cyber perpetration (SCP) subscales which encompass a range of different behaviours, including gender- and sexuality-based harassment, the exchange of pornography, and unwanted sexual requests. Analysing all these conducts within the same dimension may result in a lack of differentiation between these different types of harassment. However, this lack of differentiation has already been reported in other studies focusing on both the online context (Gámez-Guadix et al., 2022b) and face-to-face harassment (Ortega et al., 2010; Vega-Gea et al., 2016; Witkoska & Kjellberg, 2005), suggesting that during adolescence these forms of harassment co-occur frequently, most likely as a result of adolescents’ urge towards sexual exploration. Another limitation refers to the difference in the number of items in the two scales (SCP and NCSP). Whereas the SCP scale contains nine items, the NCSP scale has only three, a circumstance which likely affected the perpetration rates found. Although there is still no consensus regarding how to measure this specific form of harassment, it would be interesting for future studies to include how the information is shared (posted, shown, or forwarded), the type of content shared (intimate messages, videos, or photos) and the perpetrator’s relationship with the victim. The combination of these dimensions would enable these manifestations of harassment to be graded in terms of severity and would provide more information about the context in which NCS occurs. Although the forwarding of sexual content without consent is the most prevalent form of specific online sexual harassment among adolescents and young adults (Patel & Roesch, 2022), this study did not include other new behaviours, such as the creation of sexual content. Future studies could improve the instrument by incorporating these dimensions and also taking into account other possible forms, such as recent incidences of sexual harassment perpetrated using artificial intelligence (Flynn et al., 2022). Finally, the data collection procedures differed between Spain and Italy (in-person and online, respectively), which might have influenced the prevalence differences. Nevertheless, although supervision by scientific professionals may lack the proximity of in-person oversight in the online format, the literature confirms that the use of online or paper questionnaires is largely indifferent. Indeed, the potential differences between using both types of questionnaires have been thoroughly explored, and the found differences have been minimal or non-existent (Gwaltney et al., 2008). Future studies could corroborate the prevalence rates obtained through both data collection formats.

## 5. Conclusions

The results presented in this study demonstrate that online sexual harassment among adolescents encompasses a wide range of aggressive behaviours and expressions that, at times, are very difficult to differentiate. Moreover, NCS is confirmed as a form of online sexual harassment that is present during this developmental stage. The present study makes a significant contribution to the field of research into online sexual harassment during adolescence by presenting a new assessment tool that has been shown to be invariant not only between Spanish and Italian adolescents, but also between boys and girls. This invariance underscores the potential usefulness of the instrument for comparative studies exploring similarities and differences in online sexual harassment among adolescents. Future research could utilise this instrument to examine risk factors, consequences, and the effectiveness of interventions at a transnational level in a gender-sensitive manner.

## Figures and Tables

**Table 1:** Prevalence of items in each country.

Items	Spain (% Yes)	Italy (% Yes)
SCP1. Making obscene (‘dirty’) comments, jokes, or gestures on a Social Networking profile	12.6	19.7
SCP2. Spreading rumours about someone else’s sexual behaviour on Social Networking sites	8.1	6.4
SCP3. Calling someone a ‘faggot’, ‘lesbo’, ‘slut’ or similar name on Social Networking sites	16.0	15.3
SCP4. Showing one’s arse or other body parts in photos on Social Networking sites	10.9	5.8
SCP5. Showing or posting sexual images, pictures, messages or obscene (‘slutty’) remarks on Social Networking sites	6.5	2.8
SCP6. Sending sexual messages or drawings to another person on Social Networking sites	10.0	6.1
SCP7. Talking about sex on the Internet and Social Networking sites	31.2	24.8
SCP8. Hinting at or asking someone to post unclothed pictures of any part of their body on Social Networking sites	8.4	3.7
SCP9. Sending or showing personal pictures of yourself in a provocative attitude or showing any part of your body on Social Networking sites	12.5	5.4
NCS1. Forwarding intimate messages from other people without their consent	12.2	7.3
NCS2. Forwarding intimate photos of other people without their consent	3.9	3.9
NCS3. Forwarding intimate videos of other people without their consent	2.2	2.9

Percentages indicate the proportion of participants who reported engaging in each conduct at least once in the past two months.

**Table 2:** Results of the confirmatory factor analysis.

Model	*N*	χ^2^ (DF)	RMSEA	Lower, Upper	*CFI*	*SRMR*
Model 1	2357	313.43 (51)	0.05	0.04, 0.05	0.96	0.07
Model 2	2357	312.74 (53)	0.05	0.04, 0.05	0.96	0.07
Spain	1020	160.03 (53)	0.04	0.04, 0.05	0.97	0.07
Italy	1337	171.55 (53)	0.04	0.03, 0.05	0.96	0.08
Boys	1196	240.70 (53)	0.05	0.05, 0.06	0.95	0.09
Girls	1161	126.40 (53)	0.04	0.03, 0.04	0.98	0.06

CFAs across countries and genders were performed only with Model 2 due to the strong correlation found between the ASCP and DSCP factors in Model 1. Chi-square was significant in all CFAs at *p* < 0.001.

**Table 3:** Results of the cross-national and cross-gender invariance factor analysis.

**Cross-National**	**χ^2^** ***(DF)***	**RMSEA**	**Lower, Upper**	**CFI**	**SRMR**	**∆ RMSEA**	**∆ CFI**
Configural	331.49 (106)	0.04	0.04, 0.05	0.97	0.07		
Strict	372.47 (116)	0.04	0.04, 0.05	0.96	0.08	0.001	−0.004
**Cross-Gender**	**χ^2^** ***(DF)***	**RMSEA**	**Lower, Upper**	**CFI**	**SRMR**	**∆ RMSEA**	**∆ CFI**
Configural	371.32 (106)	0.05	0.04, 0.05	0.96	0.08		
Strict	370.96 (116)	0.04	0.04, 0.05	0.96	0.08	−0.003	0.003

Total sample = 2357. Chi-square was significant in all steps tested at *p* < 0.001.

**Table 4:** Prevalence of online sexual harassment perpetration among peers: sexual cyber perpetration, non-consensual sharing perpetration, and both.

	Cross-National		Cross-Gender	
	Spain (%)	Italy (%)	Girls (%)	Boys (%)
None	527 (51.7)	763 (57.1)	641 (53.6)	649 (55.9)
SCP	352 (34.5)	443 (33.1)	426 (35.6)	369 (31.8)
NCSP	35 (3.5)	27 (2.0)	36 (3.0)	26 (2.2)
Both	105 (10.3)	104 (7.8)	92 (7.7)	117 (10.1)

SCP = Sexual Cyber Perpetration Scale; NCSP = Non-Consensual Sharing Perpetration Scale. Cross-national, χ^2^(3) = 11.92, *p* < 0.01; Cramer’s *V* = 0.07. Cross-gender, χ^2^(3) = 8.25, *p* < 0.05; Cramer’s *V* = 0.06.

## Data Availability

The data used in the present study are part of ongoing research projects; therefore, they are not readily available due to privacy or ethical restrictions. Data supporting reported results can be solicitated to corresponding author under request.
